# Reply to: Serological response after COVID-19 vaccination, cirrhosis, and postliver transplant

**DOI:** 10.1097/HC9.0000000000000421

**Published:** 2024-05-10

**Authors:** Maria Pilar Ballester, Eva Uson, Rajiv Jalan, Gautam Mehta

**Affiliations:** 1 Department of Digestive Disease, Hospital Clínico Universitario de Valencia, Valencia, Spain; 2 INCLIVA Biomedical Research Institute, Valencia, Spain; 3 European Foundation for the Study of Chronic Liver Failure (EF Clif), Barcelona, Spain; 4 Liver Failure Group, Institute for Liver and Digestive Health, University College London, Royal Free Campus, London, UK

We thank Drs Daungsupawong and Wiwanitkit for their insightful comments^1^ on our manuscript regarding COVID-19 vaccination responses in patients with cirrhosis and postliver transplant.^2^ They shared several comments:

1. Sample size: The data reported represent one of the largest reported cohorts of COVID-19 vaccine responses in cirrhosis or patients with autoimmune liver disease (AILD). Additionally, these data augment the existing literature for liver transplant (LT) recipients. Although the target sample size could not be reached due to the speed of the vaccination program, a total of 767 participants were included.
2. Control group: The study did include a control group of 51 healthy individuals. In fact, the primary aim was to determine if patients with chronic liver disease mount comparable humoral immune responses to healthy controls following COVID-19 vaccination. Data shows that SARS-CoV-2 Spike IgG levels were lower in patients with prior LT compared with healthy controls and other groups.
3. Particular vaccines: The effect of particular vaccines was addressed in several analyses, both by categorization according to vaccine types (mRNA, viral, or heterologous) and by comparing individual vaccine brands. In patients with AILD, the major variable associated with humoral response was the mRNA vaccine type. Additionally, the Janssen adenoviral vaccine showed a lower IgG Spike response compared to Pfizer-BioNTech.
4. Booster dosing: We completely agree that investigating the efficiency of booster dosing and the long-term stability of immune responses may yield insightful results. Indeed, we are working on these aspects, which are the main aims of the next stage of the COBALT project.
5. Other immunological markers: We did include an extensive panel of inflammatory markers to evaluate the risk of breakthrough COVID-19 infection, showing the novel finding that CD40 ligand is protective against breakthrough infection in patients post-LT. These immunological markers were also included in multivariable analyses of IgG Spike vaccine response. Nonetheless, recurrent infections were not collected, and whether these markers can be used to predict them was not analyzed in the study.
6. Prior asymptomatic COVID-19 infections: To avoid bias due to the inclusion of subjects likely to have had asymptomatic COVID-19 before recruitment, participants with serum concentrations of anti-SARS-CoV-2 Nucleocapsid IgG antibodies ≥ 5000 U were excluded from subsequent analyses. This cutoff was suggested by the kit’s manufacturer as the optimal discriminant anti-N IgG threshold by area under the receiver operating characteristic analysis, comparing pre-COVID negative serum samples to PCR-confirmed COVID-positive serum samples within 7–14 days of infection.
7. Genetic makeup: We agree with the authors that the individual’s genetic makeup may influence vaccine responses and risk of infection. This is a new line of research for future studies.
8. Defined follow-up: The study did include a defined follow-up: 8 months after the second vaccination dose or until the third vaccine dose was administered, whichever was sooner.
9. Confounding factors: Some confounding factors such as sampling time postvaccination, race, ethnicity, alcohol and tobacco consumption, and comorbidities were included. Socioeconomic status and access to health care were not considered, but all participants lived in comparable European countries with freely available health care.
